# Gas Sensing Properties of Pt- and Rh-Decorated InS Monolayer Towards Toxic Industrial Gases: A First-Principles Study

**DOI:** 10.3390/molecules30234510

**Published:** 2025-11-22

**Authors:** Jinyan Li, Junxian Lin, Shuying Huang, Dejian Hou, Shaomin Lin, Jianhong Dong

**Affiliations:** 1 School of Chemical and Environmental Engineering, Hanshan Normal University, Chaozhou 521041, China; jinyanli@hstc.edu.cn (J.L.); 202301017219@stu.hstc.edu.cn (J.L.); 202301017232@stu.hstc.edu.cn (S.H.); lsm678@hstc.edu.cn (S.L.); 2 School of Materials Science and Engineering, Hanshan Normal University, Chaozhou 521041, China

**Keywords:** InS monolayer, toxic industrial gases, adsorption, first-principles calculations, gas sensing properties

## Abstract

The development of highly sensitive gas sensors for toxic industrial gases (TIGs) is paramount for environmental monitoring and public safety. Here, the first-principles calculations were employed to systematically investigate the potential of Pt- and Rh-decorated InS (Pt-InS and Rh-InS) monolayers as advanced gas sensing materials for the five TIGs (SO_2_, NH_3_, NO, CO, and NO_2_). The results reveal that Pt and Rh atoms can be stably anchored at the InS monolayer, inducing significant modulation of its electronic properties. The Pt-InS system exhibits strong chemisorption of NH_3_ and CO, while the other TIGs interact via physisorption. In contrast, the Rh-InS monolayer demonstrates strong chemisorption and distinct electronic responses to all five gases, driven by robust hybridization between the Rh-d and TIG-p orbitals. Based on comprehensive analyses of sensitivity and recovery time, Rh-InS is identified as a theoretically promising candidate for a reusable SO_2_ sensor at room temperature, boasting a calculated rapid theoretical recovery time of 2.20 s. The Pt-InS system, conversely, shows potential for high-temperature NH_3_ sensing. Our findings highlight the exceptional and tunable gas sensing capabilities of Pt- and Rh-decorated InS monolayers, offering a theoretical foundation for designing InS-based sensing devices.

## 1. Introduction

The accelerating pace of global industrialization has led to a significant increase in the emission of toxic industrial gases, including SO_2_, NO_x_, NH_3_, and CO, from manufacturing processes and fossil fuel combustion [1,2]. These emissions pose a severe threat to both environmental integrity and human health [3,4,5]. As primary contributors to acid rain, photochemical smog, and the greenhouse effect, TIGs can also inflict irreversible damage on the respiratory, nervous, and cardiovascular systems, even at low concentrations [6]. Consequently, it is necessary to develop advanced gas sensors for the real-time, highly sensitive, and repeatable TIG detection [7,8]. Although conventional semiconductor metal oxide (MOS) gas sensors based on materials like SnO_2_ and ZnO are widely used, their practical application is limited by high operating temperatures (typically > 200 °C) [9,10]. This requirement leads to substantial energy consumption and often compromises sensitivity. To overcome these limitations, research has shifted towards two-dimensional (2D) materials [11,12]. Since the discovery of graphene, 2D materials have been identified as ideal platforms for developing next-generation, high-performance gas sensors due to their unique properties, such as atomic-scale thickness, exceptionally high specific surface area, abundant surface-active sites, and highly tunable electronic characteristics [13,14].

Recently, the InS monolayer, a member of the transition metal dichalcogenides (TMDCs) family [15,16], has garnered considerable attention in the fields of spintronics [17], photocatalysis [18], and gas sensors [19], owing to its moderate band gap, excellent chemical stability, and unique electronic structure [20,21,22,23,24]. For example, Politano et al. [19] employed density functional theory (DFT) calculations in combination with experimental techniques to demonstrate that InS nanosheets exhibit outstanding sensing performance for NO_2_, achieving a detection limit as low as 180 ppb at an operating temperature of 350 °C. Nevertheless, the adsorption and sensing mechanisms of NO_2_ on InS monolayer at the atomic scale remain elusive, and the relatively weak adsorption strength poses a significant challenge for practical applications. To address this limitation, surface functionalization via decoration with noble metals (e.g., Pt, Pd, Au, and Rh) has emerged as an effective strategy for enhancing the performance of 2D materials such as MoS_2_ [25], WSe_2_ [26], and ZrSSe [27]. While studies on various functionalized TMDCs exist, a systematic understanding of how different noble metals influence the sensing properties of the unique InS monolayer is still lacking. Pt and Rh are particularly interesting due to their renowned catalytic activity and strong interactions with small molecules [28,29]. However, a direct comparison of their effects on a single substrate for a broad range of TIGs has not been thoroughly investigated. Therefore, the integration of Pt and Rh with InS monolayers for the systematic detection of TIGs remains a critical and unexplored area. Furthermore, the underlying physicochemical mechanisms governing such hybrid systems have not yet been elucidated.

Herein, first-principles calculations were conducted to systematically evaluate and compare the gas-sensing performance of Pt- and Rh-decorated InS monolayers (Pt-InS and Rh-InS) toward five TIGs (SO_2_, NH_3_, NO, CO, and NO_2_). Both Pt and Rh atoms are found to anchor strongly to the InS monolayer, leading to substantial modulation of its electronic structure. The adsorption of each gas on Pt-InS and Rh-InS is then examined in detail through the analyses of adsorption energies, charge transfer, projected density of states, and charge density difference, which aims to elucidate the underlying interaction mechanisms between the TIGs and the doped InS monolayers. Furthermore, the sensitivity and recyclability of Pt-InS and Rh-InS monolayers to the TIGs are assessed by analyzing the variations in band gap and work function, as well as the estimated recovery times. This study not only underscores the remarkable potential of Pt-InS and Rh-InS monolayers for TIG detection but also provides a theoretical framework for the rational design of high-performance InS-based gas sensors.

## 2. Results and Discussion

### 2.1. Structural and Electronic Properties of Pt-InS and Rh-InS Monolayers

**Figure 1:**
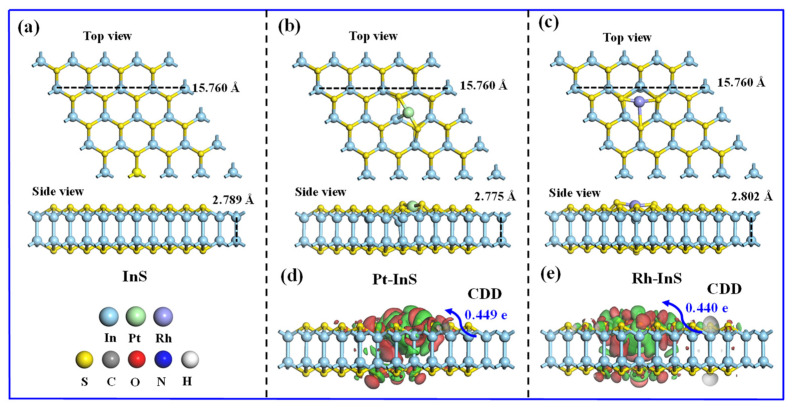
(**a**–**c**) Atomic configurations and (**d**,**e**) charge density difference (CDD) of pristine InS, Pt-InS, and Rh-InS monolayers after full relaxation.

**Figure 2:**
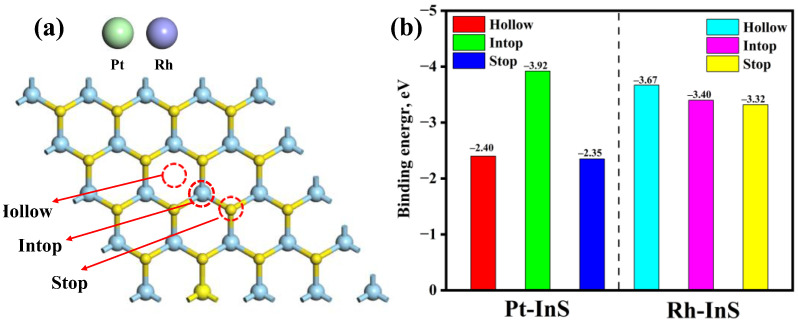
(**a**) The potential doping sites of InS monolayer for TM (TM = Pt, Rh) atoms and (**b**) their corresponding binding energy.

The InS monolayer exhibits a hexagonal lattice structure composed of In and S atoms, with each In atom covalently bonded to three adjacent S atoms, as shown in Figure 1. The calculated lattice constant of pristine InS is 3.94 Å, while the In–S and In–In bond lengths are 2.57 Å and 2.82 Å, respectively, all of which are consistent with previous reports [17,30]. To identify the preferred doping sites for Pt and Rh atoms, three potential adsorption sites, including Intop, Stop, and Hollow, were considered, as illustrated in Figure 2. As shown in Figure 2b, all configurations exhibit negative binding energies, indicating that the doping processes are exothermic and spontaneous. Notably, Pt doping at the Intop site yields the most negative binding energy (−3.92 eV), while Rh doping at the Hollow site results in the most negative value (−3.67 eV). Thus, Pt and Rh atoms preferentially adsorb at the Intop and Hollow sites, respectively. Furthermore, charge density difference (CDD) plots reveal that the doped Pt and Rh atoms act as electron acceptors, acquiring approximately 0.449 e and 0.440 e from the InS monolayer, respectively. This substantial charge transfer between Pt, Rh atoms, and InS monolayer further contributes to the superior stability of the doped systems.

**Figure 3:**
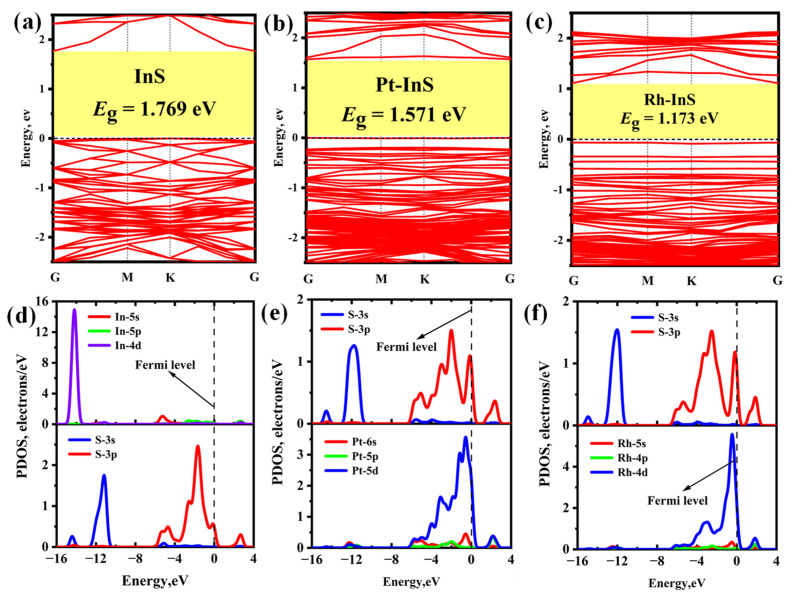
(**a**–**c**) Band structure and (**d**–**f**) projected density of state (PDOS) of (**a**,**d**) InS, (**b**,**e**) Pt-InS, and (**c**,**f**) Rh-InS monolayers.

To elucidate the electronic properties of the pristine and decorated InS monolayers, the band structures and projected density of states (PDOS) are calculated (Figure 3). The pristine InS monolayer is an indirect semiconductor with a bandgap (*E*_g_) of 1.769 eV. Upon decoration, the monolayers retain their semiconductor characteristics; however, their bandgaps are consistently reduced. For Pt-InS (Figure 3b), the *E*_g_ is reduced to 1.571 eV, while Rh decoration induces a more pronounced reduction, with the *E*_g_ narrowing to 1.173 eV (Figure 3c). This tunable bandgap engineering is crucial for optimizing the electronic response in sensing applications. Additionally, the underlying mechanism for structural stability is clarified by the PDOS analysis, as shown in Figure 3d–f. In the pristine InS monolayer, the S-3p orbital hybridizes with the In-5s orbital in the range of −6.00 eV to −3.00 eV, and with the In-5p orbital from −3.00 eV up to the Fermi level, resulting in the formation of In–S covalent bonds. In the Pt-InS and Rh-InS monolayers, the Pt-5d and Rh-4d orbitals exhibit strong overlap with the S-3p orbital across the range of −8.00 eV to 4.00 eV, with pronounced resonance peaks near the Fermi level. This suggests the formation of robust Pt–S and Rh–S covalent bonds in the doped systems, underscoring their excellent structural stability.

Although this study is theoretical, the experimental synthesis of Pt-InS and Rh-InS monolayers is considered highly feasible. High-quality InS monolayers can be fabricated using established techniques such as chemical vapor deposition. Decoration with single Pt or Rh atoms can subsequently be achieved through methods including atomic layer deposition, wet-chemical impregnation followed by reduction, or physical vapor deposition at low flux. A crucial consideration for practical applications is the stability of these single-atom decorations, particularly under sensor operating conditions. Our first-principles calculations provide strong theoretical evidence for their robust stability. The calculated high binding energies suggest strong chemical anchoring of the metal atoms to the InS substrate, resulting in a significant energy barrier against surface diffusion and subsequent aggregation. Therefore, both Pt-InS and Rh-InS systems are predicted to retain their single-atom dispersion under real operating conditions, although long-term stability will ultimately require experimental validation.

### 2.2. Adsorption of TIGs on Pt-InS Monolayer

**Figure 4:**
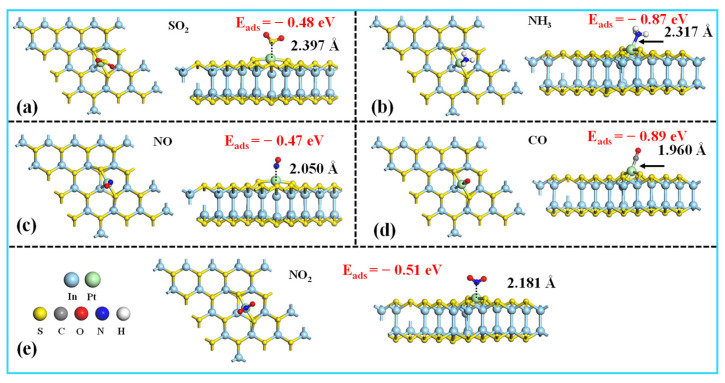
Top and side views of the most stable structures for the (**a**) SO_2_, (**b**) NH_3_, (**c**) NO, (**d**) CO, and (**e**) NO_2_ adsorbed on the Pt-InS monolayer.

To assess the gas sensing capabilities, we systematically investigated the adsorption behaviors of SO_2_, NH_3_, NO, CO, and NO_2_ on the Pt-InS surface. In our calculations, only the active transition metal (TM) atoms were considered as potential adsorption sites, and various adsorption orientations were explored. The most energetically favorable configurations are presented in Figure 4. CO and NH_3_ exhibit the strongest binding affinities to the Pt-InS surface, with adsorption energy values of −0.89 eV and −0.87 eV, respectively. As illustrated in Figure 4d, the CO molecule adsorbs atop the Pt atom through its carbon end, resulting in a short Pt–C bond length of 1.960 Å. Similarly, NH_3_ adsorbs via its nitrogen atom, with a Pt–N distance of 2.317 Å (Figure 4b). These high adsorption energies and short bond lengths indicate a strong chemisorption process. In contrast, NO, NO_2_, and SO_2_ show weaker interactions with the Pt-InS surface. The *E*_ads_ values for NO, NO_2_, and SO_2_ are −0.47 eV, −0.51 eV, and −0.48 eV, respectively, with corresponding interaction distances of 2.050 Å (Pt–N for NO), 2.181 Å (Pt–N for NO_2_), and 2.397 Å (Pt–S for SO_2_). Therefore, the adsorption of NO, NO_2_, and SO_2_ is classified as physical adsorption.

**Figure 5:**
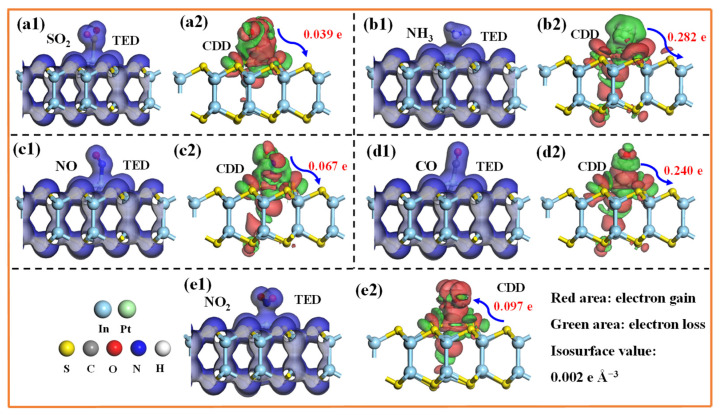
(**a1**–**e1**) Total electron density (TED) and (**a2**–**e2**) charge density difference (CDD) plots of the adsorbed Pt-InS monolayers with (**a1**,**a2**) SO_2_, (**b1**,**b2**) NH_3_, (**c1**,**c2**) NO, (**d1**,**d2**) CO, and (**e1**,**e2**) NO_2_.

To elucidate the microscopic interactions between the TIGs and the Pt-InS monolayer, we calculated the total electron density (TED) and CDD for each system (Figure 5). All gas molecules exhibit substantial electron sharing with the Pt-InS surface, as indicated by the blue regions between them in Figure 5(a1–e1), suggesting relatively strong interactions. However, the CDD plots reveal notable differences among the gases, as shown in Figure 5(a2–e2). Specifically, SO_2_, NH_3_, NO, CO act as electron donors, transferring approximately 0.039 e, 0.282 e, 0.067 e, and 0.240 e to the Pt-InS monolayer, respectively. In contrast, NO_2_ behaves as an electron acceptor, withdrawing about 0.097 e from the Pt-InS surface. Furthermore, the electron transfer values for NH_3_ and NO are significantly higher than those for the other gases, indicating a much stronger affinity of the Pt-InS monolayer for NH_3_ and NO. This observation is consistent with the previously discussed adsorption energy analysis.

**Figure 6:**
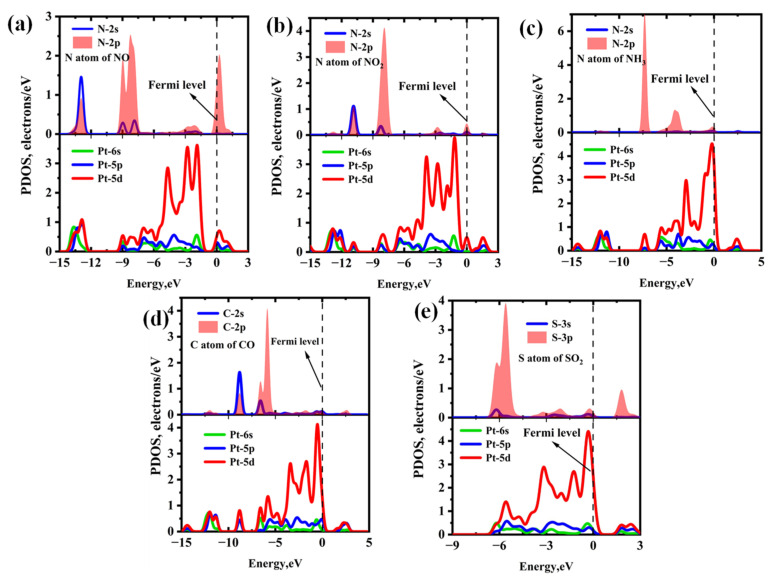
Projected density of states (PDOS) of different adsorption systems: (**a**) NO@Pt-InS, (**b**) NO_2_@Pt-InS, (**c**) NH_3_@Pt-InS, (**d**) CO@Pt-InS, and (**e**) SO_2_@Pt-InS.

**Figure 7:**
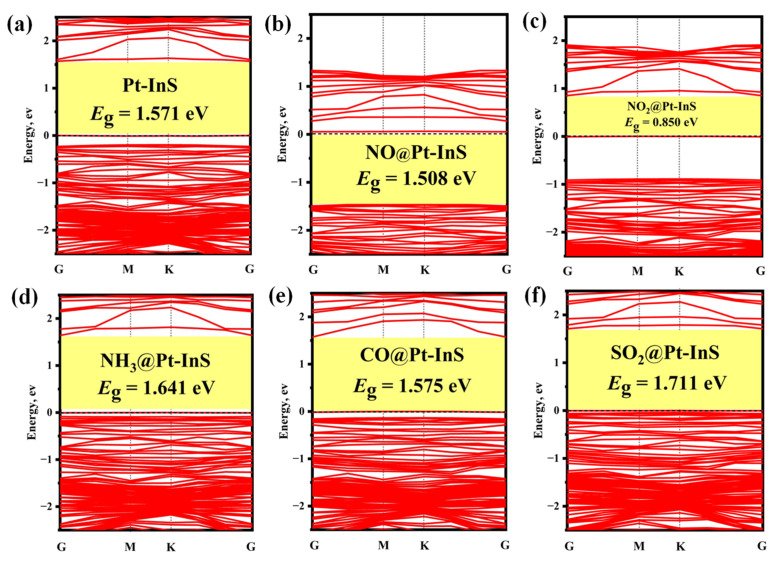
Band structures of different adsorption systems: (**a**) Pt-InS, (**b**) NO@Pt-InS, (**c**) NO_2_@Pt-InS, (**d**) NH_3_@Pt-InS, (**e**) CO@Pt-InS, and (**f**) SO_2_@Pt-InS.

Figure 6 and Figure 7 present the PDOS and band structures of Pt-InS monolayer upon adsorption of various toxic gases, respectively. For the NO, NO_2_, and NH_3_ adsorption systems (Figure 6a–c), the adsorption strengths are primarily attributed to orbital hybridizations between N-2p and Pt-5d states within the energy range of −15.00 eV to 3.00 eV. Notably, the orbital overlap in the NH_3_ adsorption system is more localized and significantly stronger than that observed in the NO and NO_2_ systems, resulting in the highest adsorption strength for NH_3_ among the three gases. In the CO@Pt-InS system (Figure 6d), the PDOS reveals pronounced hybridization between the C-2p orbital and Pt-5d state, indicating the formation of a strong Pd-C covalent bond. In contrast, for the SO_2_@Pt-InS system (Figure 6e), the orbital overlap between S-3p and Pt-5d occurs within a narrow energy range of −6.50 eV to 3.00 eV, with less prominent resonance peaks compared to the other systems, which aligns with the lower adsorption energy of SO_2_. As shown in Figure 7, the adsorption of NO, NH_3_, and CO has minimal impact on the band gap and electrical conductivity of pristine Pt-InS. In contrast, SO_2_ adsorption slightly increases the band gap from 1.571 eV to 1.711 eV, indicating a potential decrease in conductivity. The most pronounced effect is observed with NO_2_ adsorption (Figure 7c), where the interaction introduces impurity states within the original band gap, resulting in a dramatic reduction in the band gap to 0.850 eV. This substantial narrowing of the band gap by approximately 46% is expected to significantly enhance the electrical conductivity of the material, suggesting a strong and readily detectable sensing response for NO_2_.

### 2.3. Adsorption of TIGs on Rh-InS Monolayer

**Figure 8:**
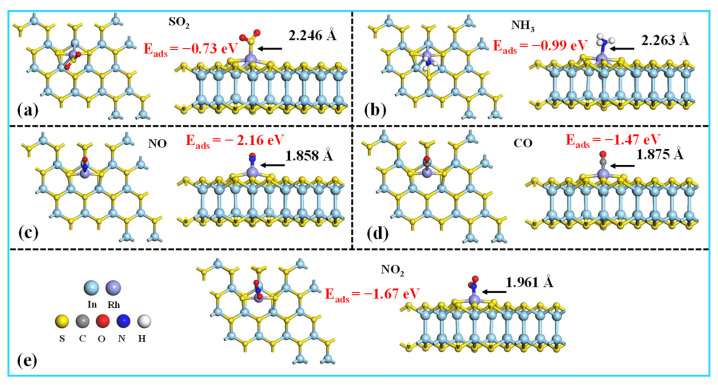
Top and side views of the most stable structures for the (**a**) SO_2_, (**b**) NH_3_, (**c**) NO, (**d**) CO, and (**e**) NO_2_ adsorbed on the Rh-InS monolayer.

**Figure 9:**
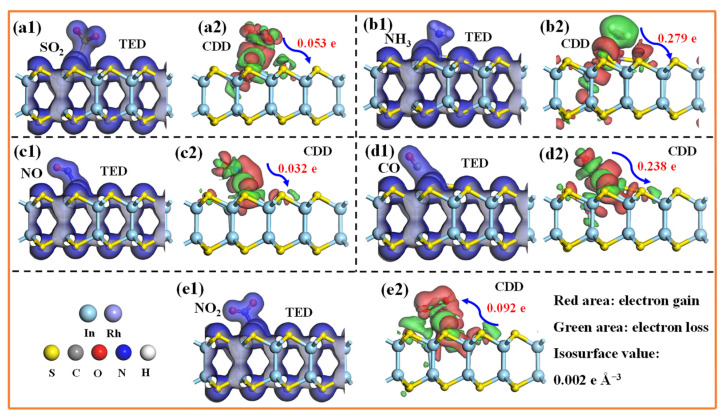
(**a1**–**e1**)Total electron density (TED) and (**a2**–**e2**) charge density difference (CDD) plots of the adsorbed Rh-InS monolayers with (**a1**,**a2**) SO_2_, (**b1**,**b2**) NH_3_, (**c1**,**c2**) NO, (**d1**,**d2**) CO, and (**e1**,**e2**) NO_2_.

Figure 8 illustrates the most energetically favorable configurations for five TIGs adsorbed on the Rh-InS monolayer. As observed, all gases preferentially bind to the decorated Rh atom. Specifically, the SO_2_ molecule adsorbs via its sulfur atom, while NH_3_, NO, and NO_2_ bind through their respective nitrogen atoms, and CO interacts via its carbon atom. The calculated adsorption energies are −0.73 eV (SO_2_), −0.99 eV (NH_3_), −2.16 eV (NO), −1.47 eV (CO), and −1.67 eV (NO_2_), with corresponding Rh-adsorbate bond lengths of 2.246 Å, 2.263 Å, 1.858 Å, 1.875 Å, and 1.961 Å, respectively. These equilibrium adsorption distances fall within the theoretical bonding ranges, indicating that chemisorption is the dominant adsorption mechanism for these gases. The strong chemical interactions between the gases and the Rh-InS substrate are further supported by the significant electron sharing observed in all five adsorption systems, as depicted in the TED plots in Figure 9(a1–e1). Additionally, the CDD plots (Figure 9(a2–e2)) visualize the spatial redistribution of electrons. SO_2_, NH_3_, NO, and CO act as electron donors, transferring approximately 0.053 e, 0.279 e, 0.032 e, and 0.238 e to the Rh-InS substrate, respectively. Conversely, the NO_2_ functions as an electron acceptor, acquiring about 0.092 e from the monolayer. The substantial charge transfer observed, particularly in the NH_3_ and CO systems, underscores the significant electronic perturbation of Rh-InS upon gas adsorption.

**Figure 10:**
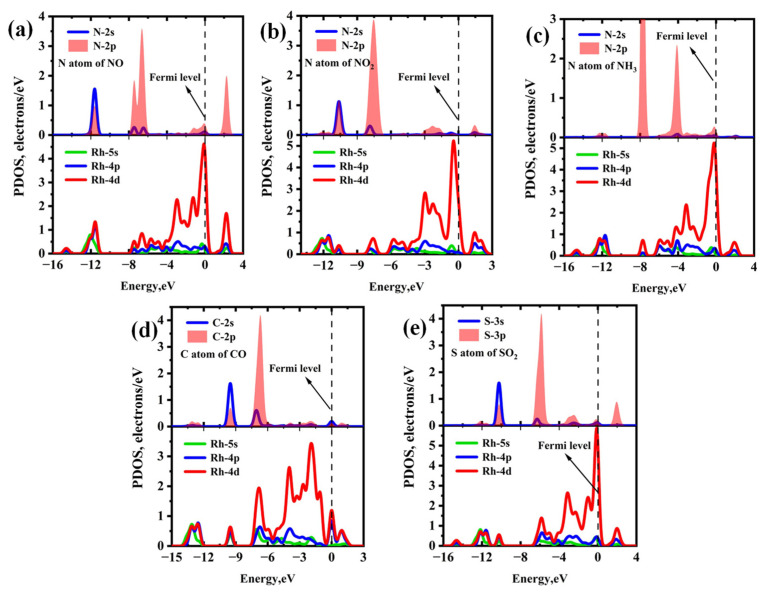
Projected density of states (PDOS) of different adsorption systems: (**a**) NO@Rh-InS, (**b**) NO_2_@Rh-InS, (**c**) NH_3_@Rh-InS, (**d**) CO@Rh-InS, and (**e**) SO_2_@Rh-InS.

**Figure 11:**
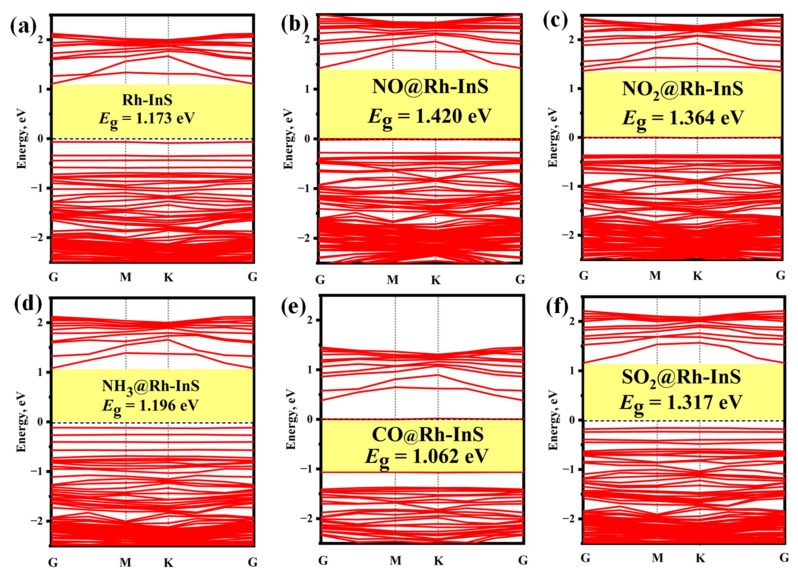
Band structures of different adsorption systems: (**a**) Rh-InS, (**b**) NO@Rh-InS, (**c**) NO_2_@Rh-InS, (**d**) NH_3_@Rh-InS, (**e**) CO@Rh-InS, and (**f**) SO_2_@Rh-InS.

The PDOS and band structure analysis for each Rh-InS adsorption system are presented in Figure 10 and Figure 11, respectively. For the nitrogen-containing species (Figure 10a–c), a significant overlap between the N-2p and Rh-4d orbitals is observed within the energy range of −13.00 eV to 3.00 eV, indicating the formation of strong Rh-N covalent bonds. In the case of CO adsorption (Figure 10d), significant hybridization occurs between the Rh-4d and C-2p orbitals in the energy interval of −10.50 eV to −5.50 eV, with two distinct hybrid peaks appearing at approximately −9.50 eV and −6.85 eV. For SO_2_ adsorption (Figure 10e), the S-3p orbital exhibits strong hybridization with the Rh-4d orbital across the entire energy range, accompanied by several resonance peaks. Additionally, compared to the bandgap of pristine Rh-InS (Figure 11a), gas interactions induce diverse and significant changes. Notably, NO adsorption induces the most substantial widening of the bandgap to 1.420 eV, whereas CO causes a marked narrowing to 1.062 eV. Other adsorbates like NO_2_ and SO_2_ also widen the bandgap, while NH_3_ has a nearly negligible effect. These electronic alterations are advantageous for enhancing the gas sensing capabilities of the Rh-InS monolayer.

### 2.4. Evaluation of InS-Based Gas Sensors

Sensitivity refers to the ability of a material to detect and respond to the presence of a specific gas analyte. The adsorption of gas molecules onto Pt-InS and Rh-InS fundamentally alters their band gap (E_g_), electrical conductivity (σ), and work function (Φ). These properties are therefore essential metrics for evaluating the sensitivity of these materials. The parameters σ and Φ are defined as follows [31,32]:

(1) $$\sigma \;=\;A\cdot \exp({-E}_{g}/2k_{B}T)$$

(2) $$\Phi =\Phi_{\mathrm{vac}}{-\;\Phi }_{\mathrm{Fer}}$$

**Figure 12:**
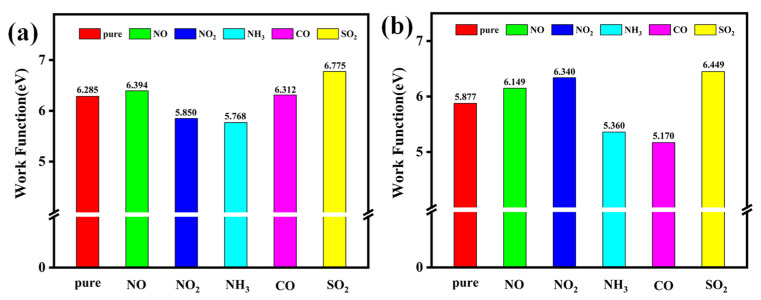
Work functions of the (**a**) Pt-InS and (**b**) Rh-InS monolayers before and after the adsorption of toxic gases.

**Figure 13:**
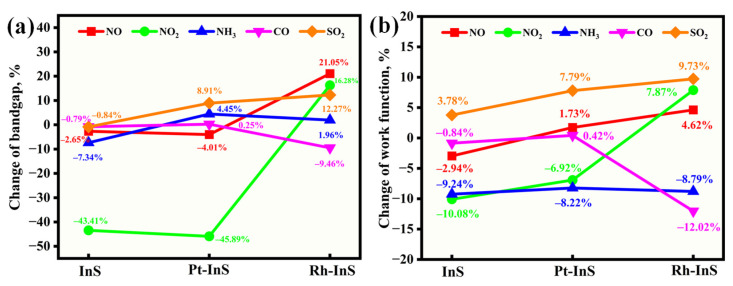
Rate of changes in (**a**) band gap and (**b**) work function for different toxic gases adsorbed on the InS, Pt-InS, and Rh-InS monolayers.

Here, A is a constant, k_B_ is the Boltzmann constant, and T denotes temperature. $\Phi_{\mathrm{vac}}$ and ${\;\Phi }_{\mathrm{Fer}}$ represent the vacuum level and Fermi level, respectively. Based on these definitions, the work functions of Pt-InS and Rh-InS monolayers, both before and after gas adsorption, are presented in Figure 12. Furthermore, Figure 13 illustrates the changes in band gap (ΔE_g_) and work function (ΔΦ) induced by the adsorption of five toxic gases.

As shown in Figure 13a, the pristine InS and Pt-InS monolayers exhibit negligible band gap modulation upon exposure to the NO, NH_3_, CO, and SO_2_ molecules, with most changes falling within ±5%. However, the adsorption of NO_2_ induces significant changes in the band gap of both InS and Pt-InS monolayers, with |ΔE_g_| values of 43.41% and 45.89%, respectively. Due to the weak adsorption strength of InS for these gases, only the Pt-InS monolayer exhibits excellent gas sensitivity toward NO_2_. In contrast, the Rh-InS monolayer exhibits an even more pronounced response, characterized by substantial band gap widening for NO (+21.05%) and NO_2_ (+16.28%). Additionally, SO_2_ and CO adsorption results in considerable |ΔE_g_| values of 12.27% and 9.46%, respectively, while NH_3_ adsorption leads to negligible band gap changes. Therefore, the Rh-InS monolayer is well-suited to function as a resistive gas sensor for the detection of NO, NO_2_, SO_2_ and CO molecules.

As illustrated in Figure 12 and Figure 13b, the Pt-InS monolayer exhibits different work function modulation, with positive changes observed for NO (+1.73%), CO (+0.42%), and SO_2_ (+7.79%). In contrast, NO_2_ and NH_3_ adsorption lead to decreases in work function (−6.92% and −8.22%, respectively). These findings suggest that the Pt-InS monolayer can serve as a work function-based gas sensor for SO_2_ and NH_3_. By comparison, the Rh-InS monolayer shows more substantial changes in work function. Specifically, SO_2_ adsorption results in a significant increase (+9.73%), while NH_3_ and CO adsorption cause decreases of −8.79% and −12.02%, respectively. The pronounced changes in work function for Rh-InS suggest strong electronic interactions and charge transfer, indicating that the Rh-InS monolayer exhibits high sensitivity and holds great promise as a work function-based gas sensor for the detection of SO_2_, NH_3,_ and CO.

**Figure 14:**
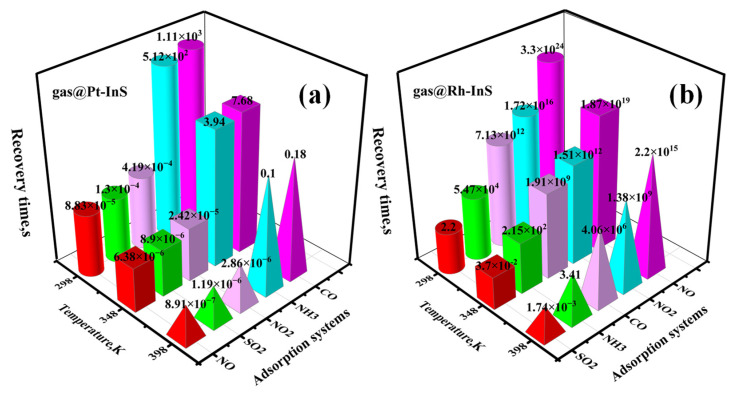
Recovery times for different gases adsorbed on (**a**) Pt-InS and (**b**) Rh-InS monolayers at various temperatures.

Recovery time is another critical parameter that determines how quickly a sensor returns to its initial state following the removal of the target gas. According to transition state theory, the recovery time (τ) can be expressed as [33]

(3) $$\tau ={{\;\upsilon }_{0}}^{-1}\exp(-E_{ads}/k_{B}T)$$

where ${\;\upsilon }_{0}$ is the attempt frequency (10^12^ s^−1^), *E*_ads_ is the adsorption energy, and *T* is the operating temperature (K). Figure 14 presents the recovery times for the desorption of toxic gases from the Pt-InS and Rh-InS surfaces at various temperatures.

For the Pt-InS monolayer (Figure 14a), the recovery times for NO, NO_2_, and SO_2_ at room temperature (298 K) are 8.83 × 10^−5^ s, 4.19 × 10^−4^ s, and 1.3 × 10^−4^ s, respectively. Such extremely short contact times between these gases and Pt-InS are insufficient to generate detectable signals. Conversely, CO and NH_3_ exhibit exceptionally prolonged recovery times, reaching approximately 1.11 × 10^3^ s and 5.12 × 10^2^ s at 298 K, suggesting that the two gases are difficult to timely desorb from the Pt-InS surface. When the temperature is raised to 348 K, Pt-InS shows excellent reusability for CO and NH_3_, with recovery times reduced sharply to 7.68 s and 3.94 s, respectively. For the Rh-InS monolayer (Figure 14b), the recovery times for NO, NO_2_, NH_3_ and CO are exceedingly long at 298 K, ranging from 5.47 × 10^4^ s to 3.30 × 10^24^ s, reflecting strong binding interactions that significantly impede rapid desorption. Even at 398 K, the recovery times remain substantial, except for the NH_3_@Rh-InS system, which achieves a recovery time of 3.41 s. Notably, Rh-InS exhibits a moderate recovery time of approximately 2.20 s for SO_2_ at 298 K. In conclusion, Rh-InS is a promising candidate for a reusable sensing material for SO_2_ at room temperature. It is important to note that this prediction is based on calculated recovery times in an ideal system, and experimental validation would be necessary to account for kinetic effects and environmental factors in real-world applications. In contrast, Pt-InS is better suited as a work function-based gas sensor for NH_3_ at high temperature (348 K).

## 3. Conclusions

In this study, the potential of Pt-InS and Rh-InS monolayers as high-performance gas sensors for five TIGs was systematically investigated using first-principles calculations. The electronic properties of each adsorption system, including the PDOS, CDD, band structures, and work function, were comprehensively analyzed. The key conclusions are summarized as follows.

(1) Pt and Rh atoms can be stably anchored onto the InS monolayer at the Intop and Hollow sites, exhibiting substantial binding energies of −3.92 eV and −3.67 eV, respectively. Furthermore, the incorporation of Pt and Rh significantly reduces the band gap and enhances the electrical conductivity of the pristine InS monolayer.

(2) NO, NO_2_, and SO_2_ are physically adsorbed on the Pt-InS monolayer, whereas CO and NH_3_ undergo chemisorption. In contrast, all five gases interact with the Rh-InS monolayer via chemisorption. The underlying microscopic mechanisms are elucidated through analyses of orbital hybridization.

(3) The Pt-InS monolayer demonstrates exceptional sensitivity toward NO_2_, attributed to the introduction of impurity states within the band gap upon NO_2_ adsorption. In comparison, the Rh-InS monolayer exhibits broad sensing capabilities for NO, NO_2_, SO_2_ and CO, as evidenced by significant changes in both band gap and work function.

(4) Recovery time analysis indicates that, based on theoretical calculations, the Rh-InS monolayer is a promising potential reusable sensor for SO_2_ detection at room temperature, featuring a calculated rapid recovery time of 2.20 s. Conversely, the Pt-InS system is more suitable as a practical sensor for NH_3_ at high temperature, enabling efficient reusability.

## 4. Computational Details

All the spin calculations in this study were performed using the DMol^3^ module within the Materials Studio 2020 package [34]. To ensure both computational efficiency and accuracy, a 4 × 4 × 1 supercell model, which is consistent with that employed in reference [22], was adopted. Following convergence testing (Figure S1), a vacuum layer of 20 Å was introduced along the vertical direction to eliminate interactions between atomic layers. During structural optimization and energy calculations, the generalized gradient approximation (GGA) with the Perdew–Burke–Ernzerhof (PBE) functional was utilized [35], in conjunction with Grimme’s DFT-D3 dispersion correction to account for van der Waals interactions [36]. The DFT semi-core pseudopotentials (DSPPs) were employed to treat the core electrons [37], and a double numerical plus polarization (DNP) basis set with an orbital cutoff radius of 5.2 Å was applied. The DIIS iteration number was set to 6 [38], and a smearing value of 0.005 Ha was applied. The convergence criteria for geometric optimization were as follows: energy self-consistency ≤ 1.0 × 10^−5^ Ha, atomic force ≤ 2.0 × 10^−3^ Ha/Å, and maximum displacement ≤ 5.0 × 10^−3^ Ha/Å. Furthermore, Monkhorst–Pack k-point density [39] convergence tests were performed for the TM-InS systems (Figure S1), confirming the use of a 4 × 4 × 1 k-point grid for structural optimization and a denser 10 × 10 × 1 grid for property calculations.

To evaluate the stability of the Pt-InS and Rh-InS monolayers, the binding energy (*E*_bin_) at different doping sites was calculated according to the following equation [12]:

*E*_b_ = *E*_TM-InS_ − *E*_InS_ − *E*_TM_ (4)

where *E*_TM-InS_, *E*_InS_, and *E*_TM_ are the total energies of the TM-doped InS system, the pristine InS, and an isolated TM atom (TM = Pt, Rh), respectively. To further analyze the gas adsorption behavior of the TM–InS system, the adsorption energy (*E*_ads_) was calculated as follows [40,41]:

*E*_ads_ = *E*_gas@TM-InS_ − *E*_TM-InS_ − *E*_gas_ (5)

where E_gas@TM-InS_ is the total energy of the TM–InS system with gas adsorption. E_TM-InS_ and E_gas_ are the total energies of the clean TM–InS and isolated gas molecule, respectively. Generally, a larger absolute value of *E*_ads_ indicates a stronger adsorption effect.

Charge transfer (Δ*Q*) in this work was computed using the Mulliken population analysis method [42], which is widely used for its simplicity in analyzing electron density distributions. The amount of Δ*Q* is given by [43]

Δ*Q* = *Q*_adsorbed-gas_ − *Q*_free-gas_ (6)

where *Q*_adsorbed-gas_ and *Q*_free-gas_ represent the charges of a single gas molecule after and before adsorption, respectively. A positive value of Δ*Q* indicates that electron transfers from the gas molecule to the TM–InS surface, whereas a negative value signifies that electron transfers from the TM–InS system to the gas molecule.

## Data Availability

The data presented in this study are available on request from the corresponding author.

## Supplementary Materials

The following supporting information can be downloaded at: https://www.mdpi.com/article/10.3390/molecules30234510/s1, Figure S1: Convergence tests for computational parameters. (a) Adsorption energy of NH_3_ on the Pt-InS monolayer as a function of the vacuum layer thickness, (b) total energy of a 4 × 4 × 1 InS monolayer supercell as a function of the k-point mesh.
