# Supplementary material for: Gas Sensing Properties of Pt- and Rh-Decorated InS Monolayer Towards Toxic Industrial Gases: A First-Principles Study
Source: Molecules. 2025 Nov 22;30(23):4510. doi: 10.3390/molecules30234510 (PMC12693120; doi:10.3390/molecules30234510)
Supplement: Supplementary file 1 [file molecules-30-04510-s001.zip › molecules-3960145-supplementary.pdf]

Supporting Information

Gas Sensing Properties of Pt- and Rh-Decorated InS Monolayer towards Toxic Industrial Gases: A First-Principles Study

Jinyan Li [a b], junxian Lin [a b], Shuying Huang [a b], Dejian Hou [a b], Shaomin Lin [a b], Jianhong Dong [a b *]

[a)] School of Chemical and Environmental Engineering, Hanshan Normal University, Chaozhou, 521041, China

[b)] School of Materials Science and Engineering, Hanshan Normal University, Chaozhou, 521041, China

Content:

Figure S1. Convergence tests for computational parameters. (a) Adsorption energy of NH_3 on the Pt-InS monolayer as a function of the vacuum layer thickness, (b) total energy of a 4×4×1 InS monolayer supercell as a function of the k-point mesh.

[*] Corresponding author.

E-mail address: dongjh@hstc.edu.cn.

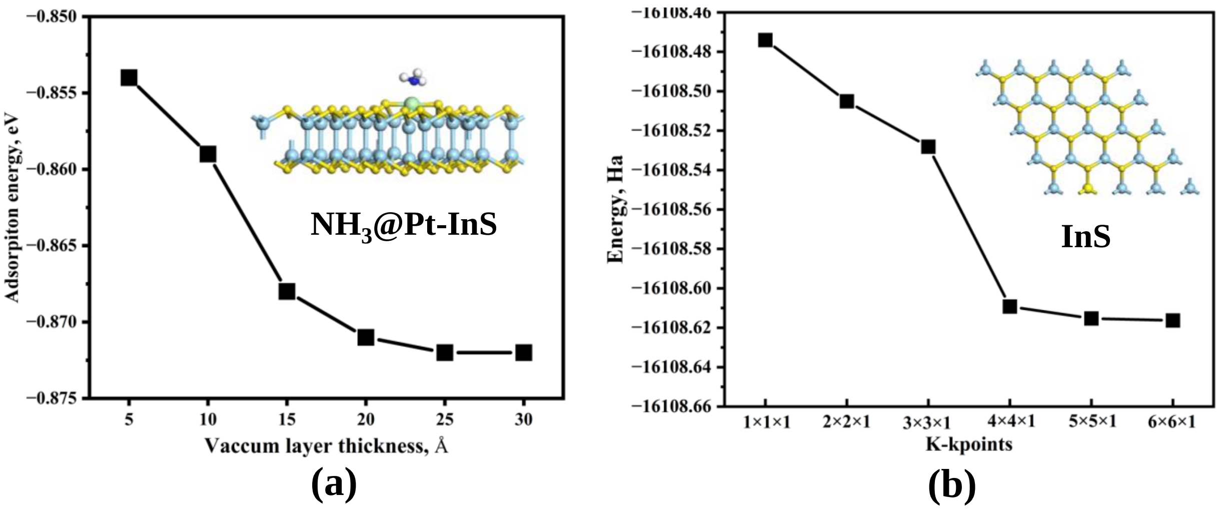

Figure S1. Convergence tests for computational parameters. (a) Adsorption energy of NH_3 on the Pt-InS monolayer as a function of the vacuum layer thickness, (b) total energy of a 4×4×1 InS monolayer supercell as a function of the k-point mesh.
